# Supplementary material for: Are carbon emissions trading and green financial instruments synergistic? -Comprehensive quantitative research based on content analysis
Source: PLoS One. 2024 Mar 7;19(3):e0298601. doi: 10.1371/journal.pone.0298601 (PMC10919601; doi:10.1371/journal.pone.0298601)
Supplement: S1 Data — (ZIP) [file pone.0298601.s001.zip › Data/地级市Z_F工作报告.zip!/╣≤╓▌/┴∙┼╠╦«╩╨/└┤╫╘╣1⁄2╓┌║┼í╛┬φ┐╦╩2╛▌═°í┐_2014.docx]

各位代表：

　　现在，我代表市人民政府向大会作工作报告，请予审议，并请市政协委员和其他列席人员提出意见。

　　2013 年工作回顾过去一年，在市委的坚强领导下，在市人大、市政协的监督和支持下，我们始终坚持主基调，实施主战略，以承办省第八届旅发大会为契机，以“5 个100 工程”为抓手，紧紧依靠全市各级干部和群众，坚定科学发展、后发赶超、同步小康的信心和决心，锐意进取、奋力拼搏、扎实苦干，全市经济社会发展呈现出“增速较快、结构趋优、位次前移、民生改善”的良好态势。

　　一年来，我们主要做了以下八个方面的工作。

　　——坚持凝心聚力抓经济，发展形势不断向好。努力克服经济下行压力和安全生产严峻形势，经济保持了平稳健康运行。全市生产总值完成882.11 亿元，增长15.9%，人均生产总值突破3 万元； 财政总收入完成178.31 亿元，公共财政预算收入完成123.59 亿元，分别增长9.88%和19.02%；全社会固定资产投资完成1480亿元，增长35.9%，其中50万元以上固定资产投资突破千亿元大 关，完成1054.93 亿元，增长37.7%；社会消费品零售总额完成209.33 亿元，增长14.5%；城镇居民人均可支配收入达19620 元； 农民人均纯收入达5934 元，增长14.5%；金融机构存、贷款余额分别为754.42亿元、599.88 亿元，分别增长11.57%、18%。全面小康实现程度达73.4%，提高4 个百分点。全省增比进位预排名， 我市从2012 年第5位上升至第3 位，盘县进入西部十强县和全国百强县，填补了贵州全国百强县的空白。

　　——坚持创新驱动促转型，经济结构不断优化。围绕农民增收致富抓农业转型，完成农林牧渔业增加值58.06亿元，增长6.5%。粮经比由63:37 调整到51:49。粮食总产量达80.9 万吨， 增长7.45%。建成红心猕猴桃、茶叶、红花油茶等特色产业示范基地25 万亩。市级以上农业产业化龙头企业达82 家，农民专业合作经济组织达549个。12个现代高效农业示范园区建设加快推进。围绕优化结构抓工业升级，完成500万元以上工业增加值433 亿元，增长20.4%；新增规模以上工业企业83户。主要工业产品产量持续增长，完成原煤产量7309万吨、洗精煤2526.15万吨、焦炭562.94万吨、钢材454.55万吨、水泥720.8万吨、发电量421.75亿千瓦时；工业用电量122.55亿千瓦时。煤矿企业兼并重组工作有序推进。黔桂天能130万吨焦化、盘北煤矸石发电厂1号机组等一批重大产业项目建成投产。实施产业园区“百日攻坚行动”， 完成园区基础设施投资110亿元、产业项目投资220亿元；建成标准厂房180 万平方米。淘汰落后产能207.4万吨，单位生产总 值能耗下降3.42%，主要污染物排放总量控制在省下达指标范围内。围绕现代服务业抓三产优化，完成三产增加值320.26亿元， 增长16%。旅游业加快发展，接待游客700 万人次，实现旅游收入44.36 亿元，增长34.9%。凉都会展中心投入使用，建成盘江雅阁、凉都锦江2个五星级酒店，五金商贸城、西南家居博览城等一批商贸物流项目相继建成。坚持把“5 个100工程”作为转型发展的重要抓手，建设了一批重点项目，打造了一批亮点工程。

　　——坚持办好旅发促发展，城市品质不断提升。举全市之力，集全市之智，突出“创新业态，转型发展，打造六盘水旅游产业升级版”的主题，办成了一次“圆满、精彩、出彩”的旅发大会。坚持一地举办、全市联动，借势发力、借题发挥，无中生有、小题大做，以旅发大会助推城镇建设，实施了市中心城区和盘县、六枝特区县城城市基础设施和功能性市政设施等一批重点工程， 城市面貌焕然一新，城市品质大幅提升。规划建设了一批业态新、环境优、容量大的城市综合体，促进新区拓展与旧城改造共同推进；启动了25个特色小城镇建设，重点打造了淤泥、羊场、岩脚、玉舍等一批示范小城镇。坚持以旅发大会提升凉都品牌，重点建设了野玉海、牂牁江、妥乐古银杏、坡上草原、陇脚月亮河、韭菜坪等特色旅游景区，玉舍森林公园被评为国家4A级旅游景区， 明湖湿地公园成为全省首个国家级湿地公园。“贵州屋脊·中国凉都”的知名度和美誉度不断提升，荣获“美丽中国·十大生态文明城市”、“中国红豆杉之乡”、“中国野生猕猴桃之乡”等称号；位列中国最具竞争力城市西部排行榜第7名、中国十佳开发潜力城市排行榜第9 名。

　　——坚持基础先行筑平台，发展条件不断改善。交通建设取得新进展，沪昆客专境内段加快推进，安六城际高铁预可研报告获得批复，六盘水火车站改造主体工程完工。水盘高速、六镇高速建成通车，六六高速、杭瑞高速毕都段、机场高速、市中心城区内环快线加快推进，盘兴高速开工建设；建成通村油路（水泥路）2262.3公里，提前两年完成“十二五”规划目标。月照机场主体工程基本完工，盘县支线机场完成规划选址工作。水利建设取得新突破，2013年全省集中开工骨干水源工程20个，我市占5个； 黔中水利枢纽工程大坝顺利封顶，白河沟、鱼洞坝、旧院水库全面竣工；治理病险水库5座；解决29.39万人农村人口饮水安全问题。生态建设力度加大，实施市中心城区108个山头绿化1.04万亩，完成营造林52.54万亩，森林覆盖率提高到43.3%；完成水土流失治理48.44平方公里、石漠化治理53.5平方公里。启动实施“四在农家·美丽乡村”基础设施建设六项行动计划，完成14.1万户民居整治。

　　——坚持投资拉动增后劲，发展基础不断巩固。紧紧围绕国发2 号文件和“5 个100 工程”谋划项目、实施项目、推进项目，完成工业投资490亿元、交通投资92.6亿元、水利投资43亿元、城市建设和房地产投资219亿元、其他投资134.3亿元，完成社会投资455 亿元。加强项目策划和包装，储备项目3518个、总投 资1.56 万亿元。坚持用项目开工“倒逼”前期工作，抓好项目建设季度集中开工，全年新开工项目1261个、总投资2251亿元，完成投资711.4亿元。坚持半年召开一次项目建设现场观摩会， 实行“旬调度、月督查、季考核”机制，项目建设进度加快，固定资产投资增速排名全省第一。积极向上争取支持，全力保障项目建设用地，新增用地指标2.7万亩。

　　——坚持改革开放增活力，内生动力不断增强。启动“营改增”试点，有序推进分税制财政体制改革。加大“引金入市”力度， 成功引进重庆银行、招商银行、海通证券、方正证券，新增村镇银行和小额贷款公司各1家。全年融资到位资金116亿元，其中市级平台公司31.71 亿元。成功发行企业债券31 亿元，市级第二期16 亿元获国家发展改革委批准。切实推进简政放权，市级取消和调整行政审批事项189 项，行政审批时限压缩率达51.3%，公安、国税、地税、工商等市级审批权限直接下放到开发区（园区）； 市级行政事业性收费由122 项减少到69 项，为3 万余户企业和个体工商户减免税6.45 亿元；全市压缩5%的行政经费共4501 万元用于支持教育事业。大力发展县域经济，县域经济占GDP 比重接近70%。全面完成民营经济三年倍增计划，民营经济增加值占GDP 比重达48%。成功举办中国凉都·六盘水休闲产业博览会、招商引资经贸洽谈会等重大活动，组织长三角、珠三角、香港、大连、昆明等系列招商推介活动，全年招商引资实际到位资金1100 亿元，增长69.2%。

　　——坚持公共服务保需求，社会事业不断进步。实施教育“9+3”计划和“四项突破工程”，中、小学生辍学率分别降至2.6%、0.3%； 完成学前教育工程14 个、高中教育工程50 个，建设农村寄宿制学校学生宿舍9.66 万平方米；农村义务教育阶段学生营养餐基本实现全覆盖；率先在全省推行“产业园区+标准厂房+职业教育”模式，完成职业教育、高中教育招生68959人。鑫晟煤化工“煤气流床气化关键技术与工业示范”项目获国家立项，实现了我市项目在国家“863”计划零的突破；启动13个省市重点实验室、工程技术研究中心和科技创新团队建设；建成国家煤炭清洁转化产品质量监督检验中心；贵州科学院六盘水分院挂牌。建成贵州三线建设博物馆、凉都体育中心、六盘水美术馆等一批标志性公共服务设施，丰富了凉都品牌内涵；成功举办第十届中国凉都消夏文化节、夏季国际马拉松赛、国际滑翔伞公开赛等活动，夏季国际马拉松赛被中国田径协会授予“马拉松铜牌赛事”称号。六枝特区原生态音乐作品《我爱我家》获第十届中国艺术节作品类“群星奖”；“公共文化服务机构队伍拓展模式”列入国家第二批公共文化服务体系示范项目。成功创建三级甲等和二级甲等医院各5家；完成19个乡镇卫生院规范化建设；新型农村合作医疗参合率达98.74%。人口计生工作主要指标实现“三降一升”。招才引智力度加大，引进高层次和急需特殊人才2503人，其中博士22 人、硕士351人。妇女、儿童、老龄、残疾人等事业稳步发展，工会、共青团、民族、宗教、外事、侨务、对台、档案、气象、 人防、红十字、地方志等工作取得新进步。

　　——坚持改善民生谋福祉，保障能力不断提高。扎实抓好“二十件民生实事”，完成投资161 亿元。新增城镇就业10.1万人，城镇登记失业率为3.95%。社会保障标准、医疗保障水平大幅提高， 城乡基本医疗保障实现全覆盖；全年发放低保金7亿元，惠及48 万城乡低保对象；完成24万人冬春困难救助。完成3.57万套保障性安居工程建设和2.8万户农村危房改造。大力实施集中连片特困地区扶贫攻坚规划，争取上级财政扶贫资金2.85亿元，启动实施扶贫生态移民搬迁2万人，减少贫困人口15.26万人，20个乡镇实现“减贫摘帽”，52个贫困村实现“整村推进”。建成省救灾物资储备库六盘水代储库、市流浪未成年人救助保护中心和市救助管理站。深入开展“平安凉都”建设，大力实施“天眼工程”和“333工程”，全市未发生重大群体性事件和恶性治安案件。安全生产形势平稳趋好，前所未有地加强了安全生产队伍建设和行业监管，安全生产保障能力不断提升，实现安全生产事故起数和死亡人数“双降”，全市煤矿百万吨死亡率降低到0.54，首次实现各类安全生产事故死亡人数控制在100 人以内。

　　过去一年，我们始终坚持把政府自身建设作为做好各项工作的重要保障。严格执行中央八项规定、省委十项规定和市委十项规定，狠抓“四风”治理。加强行政效能建设，大力减政、减负、减支。强化政务公开，市级各部门“三公”经费公开全面实施。全力推进依法行政，加强行政监察和审计监督，扎实开展“阳光 晒权”活动，勤政廉政建设取得新成绩。

　　各位代表！过去一年的成绩来之不易。这是省委、省政府和市委正确领导的结果，是市人大、市政协监督支持和积极帮助的结果，是全市各族干部群众团结奋斗和社会各界大力支持的结果。在此，我代表市人民政府，向全市各族人民，向人大代表、政协委员，向各民主党派、工商联、无党派人士和各人民团体，向驻市人民解放军、武警消防官兵、政法公安干警，向所有关心和支持我市改革开放和现代化建设的各界人士，表示衷心的感谢和诚挚的谢意！

　　在充分肯定成绩的同时，我们必须看到全市经济社会发展中存在的困难和问题，主要表现在：经济总量小，经济下行压力增大，部分企业生产经营困难；产业结构单一，发展方式粗放，资源集约化、规模化利用程度低，产业链条短，附加值低；交通基础设施落后，工程性缺水问题突出，生态环境脆弱，制约发展的瓶颈依然存在；城乡居民收入差距大，城乡二元结构、城市二元结构、农村二元结构矛盾突出，城镇化水平低，基本公共服务体系尚不完善；扶贫攻坚任务依然艰巨，贫困家庭缺乏稳定、可持续的收入来源；安全生产形势依然严峻，煤矿企业兼并重组工作仍面临挑战；政府职能转变还不到位，政府工作部门还存在工作效率不高、服务意识不强的问题。对存在的这些问题，我们将在今后的工作中用改革的精神和发展的办法来加快解决。

　　 2014 年工作目标和改革重点今年是全面深化改革的第一年，也是全面建成小康社会的关键一年。政府工作的总体要求是：以党的十八大、十八届三中全会和省委十一届四次全会精神为指导，认真贯彻市委六届五次全会的决策部署，按照“全党抓发展、全民奔小康”的思路，坚持主基调主战略，坚持稳中求进、提速转型、又好又快的总要求，牢牢守住发展、生态、安全三条底线，把改革开放贯穿于经济社会发展各个领域各个环节， 大力实施创新驱动发展、产业转型升级、生态文明建设“三大战略”， 着力保障和改善民生，努力把六盘水建成产业更加优化、环境更加优美、人民更加富裕、社会更加和谐的经济强市。

　　全市经济社会发展的主要预期目标是：生产总值增长16%；财政总收入和公共财政预算收入分别增长16%；500万元以上工业增加值增长20%；全社会固定资产投资增长35%；工业经济结构进一步优化，城镇人口比重提高到40%，民营经济比重提高到50%，县域经济比重提高到70%；社会消费品零售总额增长18%；城镇居民人均可支配收入增长15%；农民人均纯收入增长16%；人口自然增长率控制在5.4‰ 以内；城镇登记失业率控制在4.2%以内；完成省下达的节能减排指标任务。

　　各位代表！贫穷落后是我市的主要矛盾，加快发展是我市的主要任务。改革开放是推动我市经济社会提速转型、后发赶超的关键一招，搞改革没有坦途，不改革没有他途。我们要坚定不移贯彻好党的十八届三中全会、省委十一届四次全会、市委六届五次全会精神，突出战略导向、问题导向、民生导向，切实增强进取意识、机遇意识、责任意识，在更高起点上全面深化改革、扩大开放，向改革要红利、要效益，以改革开放新成效推动经济社会大发展。

　　当前要重点抓好十个领域年度改革事项的落实。（1）深化资源市场化体制改革。完善公共资源交易中心运行机制。全面推行矿业权招拍挂出让制度。完善矿产资源勘探、交易配置市场化运行体制机制。推进煤层气和瓦斯综合开发利用，实现资源市场化配置、规模化开发、循环式利用。制定实施矿产资源、土地资源“圈而不采、占而不动”治理办法。积极争取大用户直供电试点。出台煤炭资源就地转化的指导意见。（2）深化统筹城乡发展体制改革。积极争取国家和省支持我市开展资源型城市可持续发展城镇化试点，开展城乡规划“多规合一”改革。制定市中心城区、县城和建制镇全面放开落户限制的具体办法。加快水城县百车河和盘县刘官镇统筹城乡转型发展项目建设。积极稳妥构建农业经营体系，壮大集体经济，赋予农民更多财产权，制定综合改革实施方案。（3）深化生态文明建设体制机制改革。成立市生态文明建设委员会。积极争取北盘江流域纳入全省生态文明制度改革试点。完善生态文明建设考评机制、奖励扶持机制和责任追究机制。建立企业治污、治气、治渣和生态环境恢复治理的保障制度。建立殡葬秩序治理联动机制。争取进入国家生态功能区补偿改革试点。建立自然资源资产产权制度和用途管制制度。（4）深化国有企业改革。全力支持水钢、水矿、盘江等大中型省属国有企业实施产权制度改革三年行动计划。制定市属国有企业分类改革实施方案。完善国有资产管理体制和国有资本经营预算制度。推进国有企业内部用人、工资制度等改革。落实支持混合所有制经济发展的政策措施。（5）深化扶贫开发机制改革。制定精准扶贫实施意见。完善扶贫工作考核办法。建立扶贫资金有偿使用、滚动发展、多元投入机制。实行扶贫资金绩效评价机制和竞争性分配方法。（6）深化财税金融改革。优化财税结构，不断降低非税收入占比。完善财政体制，健全事权与支出责任相适应的制度。强化政府债务风险管控，建立健全规范的举债融资和债务规模控制制度。编制金融业发展规划。加大“引金入市”工作力度，建立金融组织体系和借贷、资本、债券、基金、担保等金融市场体系。（7）深化教育领域综合改革。争取我市进入国家和省设立的教改实验区，大力引进优质教育资源进入我市联动办学。推行县域内校长、教师定期交流轮岗制度，创新优秀校长、教师引进机制。推进学区化改革。全面深化职校进园区办学模式，全面实施职业院校“双证书”制度。（8）深化行政管理体制改革。完成市、县两级政府机构改革。建立行政审批绩效评估机制、责任追究机制、奖惩激励机制。继续依法依规取消和下放行政审批事项，全面推行并联审批和“一章结”、“一费清”制度。深入清理和规范行政事业性收费。继续压缩5%的行政经费用于发展教育事业。出台政府购买公共服务 的指导意见。加快推进撤县设市（区）、乡改镇、镇改办工作。（9） 深化开放型经济体制改革。出台加快开放型经济改革发展的实施意见。积极推进六盘水循环经济示范城市和攀西—六盘水经济区上升为国家战略。建立区域合作平台扶持政策、奖励制度和共建共享、收益分成机制。完善招商引资项目考核办法，对招商项目实行连续三年考核。推行招商引资项目全程代办和并联审批制度。（10）深化社会领域改革。统筹推进医疗保障、医疗服务、公共卫生、药品供应、监管体制综合改革。制定出台全市养老服务体系中长期发展规划和实施意见。组建科技成果转化后补基金。深化文化体制改革。编制城乡社区服务体系中长期建设规划，出台加强城市基层管理推进社区建设的实施意见。推进诉访分离改革。2014 年工作安排围绕实现全年目标，重点抓好以下工作。

　　（一）着力推进产业转型升级，全力打造六盘水经济升级版。按照“一产转型、二产升级、三产优化”的思路，推动三次产业转型升级、融合发展。

　　大力推进工业转型升级。改造和提升传统工业，实现资源规模化开发、集约化利用、循环式发展，不断拓宽产业幅、延长产业链、增加附加值，是我市工业转型发展的必由之路。着力推进产业园区建设。扎实开展产业园区项目建设“春季百日攻坚行动”。加快园区水、电、路、气等基础设施建设，促进城镇基础设施、公共服务设施向园区延伸和配套。推动中小企业入驻标准厂房，新建标准厂房100万平方米以上。注重园区环境保护，“贵州屋脊·中国凉都”拒绝污染项目落地。努力提高煤炭资源就地转化率。改造提升能矿产业，扎实推进煤矿企业兼并重组工作，加快“六化”矿井建设，支持煤矿企业做大做强。加快发展以煤焦化为主的精深加工。充分用好驻市大企业、科研院所的人才和技术优势，成立市煤炭产业研究中心，筑造煤炭产业发展产学研平台和人才高地。全力推进工业循环经济发展。紧紧抓住建设国家循环经济示范城市的重大机遇，加快盘北、路喜、盘南、董地等园区煤、电（焦、气）化示范基地建设。推进大湾园区装备制造精铸件项目和盘北开发区首黔项目落地开工，启动建设水城发耳煤基液体燃料项目一期工程。支持大型国有企业和紫森源等民营企业发展煤矸石综合利用、煤层气开发利用和瓦斯发电。

　　大力发展新兴产业。加快把新能源、新材料、生物医药、电子信息、节能环保、高端制造、安全产业等战略性新兴产业培育成为重要支柱产业，丰富产业业态，完善产业体系。大力推进农业结构调整。加强现代高效农业示范园区建设。注重品种品质品牌建设，发展壮大园区主导产业，提高园区配套设施、技术装备和社会化服务水平，重点加快六枝郎岱、盘县滑石、水城米箩、钟山大河等农业园区建设，确保新增1个以上市级园区进入省级重点园区，2个以上市级园区进入省级园区。加快农业特色产业发展。大力实施农业特色产业“3155工程”，完成“3155工程”规划编制。扎实推进苗木基地建设，打造具有六盘水唯一性的农业特色种苗（木）基地。组建市农科院，加强与农业院校、科研院所的合作，搭建“3155工程”的人才科技支撑平台。设立“3155工程”发展基金，市级和各县（特区、区）分别组建2 家以上农业担保公司。出台“3155工程”考核办法。推进农业产业化经营。引进、培育一批农产品精深加工龙头企业，发展壮大省级、市级龙头企业，鼓励和支持返乡大学生、乡土人才、返乡创业人员创办示范园、示范场和农副产品加工企业。大力发展农民合作社、专业大户、家庭农场，培育农村经纪人队伍，打造特色农产品信息通道。夯实现代农业发展基础。加强以基本口粮田为重点的农田水利基础设施建设，推动以农业机械为重点的农业生产工具变革。完善农产品质量和食品安全体系。大力培育适应现代农业发展要求和符合市场需求的新型职业农民，不断提升农民增收致富的能力。依法、规范、加快推进土地流转。

　　大力发展现代服务业。加快发展以旅游业和金融业为龙头的现代服务业，完成服务业增加值381 亿元，增长16%。重点建设野玉海、牂牁江、妥乐古银杏、坡上草原、韭菜坪、梭戛等一批区域性精品景区，扶持一批具有地域和民族特色的旅游商品，开发一批精品旅游线路，推动旅游地产加快发展，全力打造“贵州屋脊·中国凉都”旅游精品品牌；推动银政、银企深度合作，用好信用市场、基金市场、证券市场、信托市场的金融产品。积极发展村镇银行、小额贷款公司和农村资金互助社等小微型金融机构，创造条件支持 民间资本依法设立中小银行；依托水钢、水矿、盘江等大型国企平台，规划建设一批大型货运场站、多式联运中转站和物流枢纽、物流园区、物流中心，大力发展公路物流、铁路物流、空港物流，努力把我市打造成为西部重要物流节点城市；积极发展总部经济、会展经济，加快发展电子商务、信息服务、研发设计、动漫、文化创意、检验检测等生产性服务业，优化发展家政服务、文化娱乐、体育健身、医疗康复、养老托幼、社区服务等面向民生的生活性服务业。

　　大力推进县域经济和民营经济健康发展。认真落实省委、省政府提高县域经济比重的政策措施，重点支持盘县、钟山、水城按照中国百强县标准开展第四轮建强县工作；支持六枝加快发展，努力实现县域经济新突破。扎实推进“提高民营经济比重五年行动计划”，完善落实“3个15万元”等优惠政策，激发各类市场主体的创业热情；学习借鉴“负面清单管理模式”，清理并废除对民营企业各种形式的不合理规定，鼓励民营企业多元投资、融合发展、做大做强。

　　（二）着力扩大有效投资，扎实推进重大项目建设。坚持把扩大投资作为拉动经济增长的主要动力，保持合理投资强度，以重大项目建设带投资、扩总量、增后劲。突出投资重点。以国发2 号文件和“5个100工程”为总抓手，狠抓项目生成、审批、开工、竣工各个环节，重点实施一批重大工程、特色产业、基础设施、社会民生等项目，确保50万元以上固定资产投资完成1450亿元以上。扎实抓好411个省、市重大项目建设。做深项目前期工作。及时调整和完善项目库，确保市级项目库总投资达到1.8万亿元以上。精心策划、包装项目，提高项目申报、招商引资的有效性和成功率。完善项目推进机制。坚持以旬保月、以月保季、以季保年，实行月调度、季分析、半年总结观摩，季度集中开工项目由县区主导、每半年集中开工项目由市级主导。注重与投产项目、达产项目联动观摩相结合，实行开工、建设、投产全过程考核。强化要素保障。科学制定项目融资年度计划和三年规划；优化、整合、规范融资平台，创新融资渠道，提高直接融资比重。合理保障重点项目建设用地需求和环境容量。

　　（三）着力推进城镇化进程，提高城镇化发展质量。紧紧抓住国家大力推进新一轮城镇化机遇，以建设国际标准旅游休闲度假城市为目标，推动城乡“五个一体化”建设，走山区特色的新型城镇化发展道路。坚持高起点规划。优化城镇空间布局，促进大中小城市和小城镇协调发展，确保城镇化率提高3 个百分点以上。坚持以人为核心，市中心城区按照100 年不落后、100万以上人口规模的要求，加快推进钟山、水城、红桥同城化发展；盘县城区按照大城市规模规划建设，挖掘“文运盘县”厚重文化，推进新老城区互动发展，建成贵州最靓丽的“西大门”；六枝城区按照中等城市规模规划建设，建成连接黔中经济区和滇中经济区的现代化精品节点城市。围绕特色、绿色，建成一批“8个1”和“8+3”项目，用3年时间完成特色城镇建设。扎实有效控制“两违”建筑，全力打造“城在山中、房在林中、人在绿中”的峰丛湿地城市景观。坚持高标准建设。加快城市基础设施和配套设施建设，完善城市功能，全面提升城市的承载力、集聚力和辐射力。加快推进凤凰山、明湖等综合体建设， 每个县集中力量打造1—2 个综合体。加快城市路网建设，建成内环快线、机场高速、5 条城市连接线，启动建设乌蒙大道、红桥东路，力争年内东南环线和东北环线开工建设。大力推进城市棚户区改造，用2 至3 年时间全面改造完成。整合各类建筑公司资源和力量，成立股份制企业，提升本土建筑行业的资质和实力。坚持高水平管理。深入开展“四创”活动。加强城市精细化管理，理顺城市管理体制，推进严格执法、规范执法、文明执法。深入整治乱停乱放、乱闯乱行等不文明行为，严厉打击非法营运车辆。积极引入社会资本参与城市管理。完善网格化、信息化、数字化城市管理机制， 整合城市信息化平台资源，努力提高城市管理水平。加快推进“智慧城市”建设。

　　（四）着力加强基础设施建设，增强区域竞争优势。全力推进交通基础设施建设。实施铁路、高速公路建设三年会战，加快建设现代综合交通运输网络。力争上半年开工建设安六城际高铁；推进六盘水至纳雍、六盘水至攀枝花、盘县松河至响水运煤专线等铁路前期工作。六六高速建成通车，加快推进杭瑞高速毕都段、盘兴高速建设，力争开工纳雍至兴义高速六盘水境内段、都香高速水城至威宁段；积极争取六盘水至赫章高速、盘县至宣威高速纳入省规划。加快国道、省道公路改造，完善市、县、乡公路网建设，推进景区景 点旅游公路和重要节点公路建设。创造条件，确保月照机场上半年建成通航；加快推进盘县支线机场前期工作。大力推进水利基础设施建设。实施水利建设三大会战，着力构建现代水利体系。加快“三位一体”规划实施，确保纳入规划的34 个中小型水库在两年内全部开工建设，今年开工建设40%以上。切实抓好双桥水库建设，做好库区移民搬迁安置工作，启动相配套的水厂和管网建设。建成卡河水库等骨干水源工程，开工建设岩博、阿雨、万营等水库。实施中小河流治理项目10个，治理病险水库3座，完成地下水开采机井232口。加快实施“四在农家·美丽乡村”基础设施建设六项行动计划。坚持基础设施向乡镇以下延伸、公共服务向农村覆盖，统筹和整合扶贫开发、产业发展、生态建设资金，发挥县乡主体作用，广泛发动群众，整寨整村整乡推进，加快建设结构合理、畅通便捷的“小康路”，安全有效、保障有力的“小康水”，特色鲜明、经济美观的“小康房”，稳定可靠、智能绿色的“小康电”，宽带融合、普遍服务的“小康讯”，功能完善、文明和谐的“小康寨”。全年实施通组（自然村）人行步道200 公里，新增、恢复、改善灌溉面积7.87 万亩；建成小康房2500 套；建成10 千伏以上项目313 个、变电站21 座，新增线路1949公里；新增80个自然村通电话、49个行政村通宽带，全面完成空白乡镇邮政局（所）补建，建成37个村邮站；完成650 个自然村寨改造建设。加强电网和信息基础设施建设。构建安全高效的电力网络体系，新建一批输变电项目， 实现中心城区220 千伏电网全覆盖；加强城乡电网改造，完成电网 投资14.2 亿元。加强城乡移动通信、宽带等基础设施建设，加快实施广播电视向下延伸工程；抓住4G 网络建设机遇，提高城乡通讯网络覆盖面。

　　（五）着力抓好生态文明建设，打造生态环保宜居城市。坚决摒去“先污染、后治理，先解决温饱、后解决环保”的观念，全力保护好六盘水蓝天白云、青山绿水，让“‘贵州屋脊·中国凉都’风行天下”大放异彩。强化环境综合治理。全面开展“治水、治气、治渣、环境监督管理能力建设与法制保障”四大行动，抓好“十二件环保实事”。扎实推进污水处理系统、垃圾收运系统、生活垃圾无害化处理系统等工程建设，城市污水、生活垃圾无害化处理率分别达到76%和85%。实施资源综合利用“双百工程”，工业固体废弃物综合利用率达到48%。开展PM2.5 监测并从今年6 月起定期发布监测信息。强化环保执法，严厉查处、打击环保违法行为。加快生态工程建设。抢抓国家实施新一轮退耕还林还草政策机遇，全面实施25度以上坡耕地退耕还林工程，突出沿河、沿路、沿库、沿园、沿寨生态建设“五个优先”，加强森林资源保护和管理，打造绿色生态产业。完成营造林45 万亩以上，治理石漠化53.5平方公里、水土流失55.58 平方公里。森林覆盖率提高2 个百分点以上。加强地质环境整治和矿山生态恢复治理。推动殡葬生态文明建设， 制定出台殡葬事业中长期发展规划和实施意见。

　　（六）着力扩大对外开放，提升美丽凉都新形象。构建对外开放平台。继续办好中国凉都消夏文化节等活动。坚持借势发力、借题发 挥、借船出海，深化“请进来、走出去”措施和路径，不断提升“贵州屋脊·中国凉都”知名度和美誉度。强化“5 个100 工程”对外开放的基础平台作用。加强与中关村贵阳科技园的合作。加快推进六盘水海关、检验检疫机构建设，积极创造条件申建加工贸易承接地、出口基地。强化区域交流合作。积极融入长三角、珠三角、成渝、黔中、滇中、长株潭经济区，主动承接东部发达地区产业转移， 加强与川、黔、滇、桂周边市州的经贸合作，精准对接贵阳市产业辐射带动，提升区域合作的层次和水平。提高招商引资质量。坚持市统筹、县为主，强化党政主要领导干部招商引资第一责任人职责，实行领导招商、精准招商、以商招商，突出招商工作实效，确保招商引资到位资金达到1450 亿元以上。积极参加省组织的各类招商活动，努力招大商、选好商。认真落实重大招商引资活动成果，提高招商引资项目签约率、资金到位率、开工率和投产达产率。

　　（七）着力统筹发展各项事业，推进社会全面进步。优先发展教育。深入实施教育“9+3”计划和“四项突破工程”，加快农村寄宿制学校标准化建设，促进优质教育资源均衡配置、城乡共享。大力推进“退高进初进小”，有效解决市中心城区和县城“大班额”的实际问题。全面推广“产业园区+标准厂房+职业教育”模式，加快以朵职教园建设，提高开发区（园区）职校办学能力和水平。加快六盘水师院二期工程和六盘水职院二期工程建设，扎实做好六盘水师院迎接2015年国家教育部本科院校办学评估工作。启动实施名校、名校长、名教师、优秀班主任培育工程。加强师德师风教育，提高 教师专业化水平。积极发展民族教育、学前教育、特殊教育、继续教育。提高科技创新能力。大力实施“686”科技创新工程，提高综合科技进步水平。充分发挥我市地下矿产资源、地上生物资源优势，大力开展技术攻关，建设一批省市级重点实验室和工程技术中心、企业技术中心。用好六盘水师院、六盘水职院、贵州科学院六盘水分院、市农科所等平台，加快建立产学研协调创新机制。建立和完善人才“引进、管理、使用、评价”的科学体系，深入开展招商引才、项目引才和技术引才等专项活动，积极支持和帮助本地科技人员创新创业。强化卫生计生服务。积极推进市县乡三级卫生和计生机构整合。做好疾病预防控制，强化药品安全监管。全面实施公共卫生服务项目，完成44 所村卫生室规范化建设，抓好11个乡镇卫生院周转房建设。积极开展示范乡镇卫生院和村卫生室创建活动，提升基层医疗卫生服务能力。围绕“三降一升”目标，继续抓好人口计生基层基础和“双诚信双承诺”工作，强化优质服务，着力控制政策外生育，有效提高人口素质，实现位次前移。推动文化繁荣。注重传统优秀文化和非物质文化遗产的挖掘、保护和传承，大力弘扬“三线文化”、民族文化、历史文化，突出抓好一批文化古城、古镇、古寨、古街的开发和建设。积极支持各种形式的小微型文化企业发展。建好、管好、用好文化体育场馆，开展好国际夏季马拉松赛等活动，举办好省第五届残疾人运动会，筹办好2015年的省第九届运动会。加快发展其他事业。扎实做好民族团结和双拥优抚安置工作，加强国防动员和后备力量建设，支持军队、武警部队建设和法院、检察院工作。重视档案工作，严格档案执法，加强项目档案和民生档案管理。加强司法行政、群团组织、外事、侨务、老龄、对台、气象、人防、地方志等工作，促进各项社会事业协调发展。

　　（八）着力保障和改善民生，加强和创新社会管理。坚持把保障和改善民生作为一切工作的出发点和落脚点，抓紧抓实抓好。打好扶贫决战。大力实施精准扶贫，对贫困人口精细化管理、对扶贫资源精确化配置、对贫困农户精准化扶持，做到“六个到村到户”。整合专项扶贫、行业扶贫、社会扶贫“三股力量”，加大重点乡镇“减贫摘帽”力度，深化“千企帮村、万户结对、共奔小康”活动，实施好扶贫生态移民工程。转移贫困乡镇农民6万人，减少贫困人口14.55万人以上。扩大就业创业。启动国家级创业型城市创建工作，统筹做好高校毕业生、农村转移劳动力、就业困难人员、被征地农民、扶贫生态移民、退役军人的就业和技能培训工作。全年完成失业人员再就业3000人以上，就业困难人员再就业2500人以上。完善社会保障。继续扩大城镇居民社会养老保险和新型农村社会养老保险覆盖面，基本养老保险、基本医疗保险覆盖率提高到95%以上。推进商业保险机构参与新农合经办服务及大病补充保险工作。完善城乡居民最低生活保障增长机制。健全社会救助制度。加强防灾减灾救灾工作。采取措施努力解决市中心城区燃气供需矛盾突出问题。改善住房条件。统筹推进城镇保障房、扶贫生态移民房建设和农村危房改造，促进“三房”融合，推动城镇保障房向建制镇、工矿区、产业和教育园区延伸。完成新民居建设改造14.3万户。努力抓好住房公积金支持保障房项目贷款试点城市建设工作。创新社会管理。完善新型社区管理体制，学习借鉴“贵阳经验”，有序推进撤销街道办设立社区，推动社会治理重心下移。积极探索农村新型社区建设模式；创新群众工作机制，健全领导干部信访接待日制度和领导干部下访制度，建立健全重大决定、重大决策、重大项目社会稳定风险评估机制，扎实开展矛盾纠纷排查化解，畅通群众合理诉求渠道；深入推进“平安凉都”建设，以“天眼工程”和“333”工程为抓手， 加强人防、技防综合治安防控体系建设。深入开展好禁毒工作，落实好禁毒工作有效措施。依法严防和惩治各类违法犯罪活动，努力提升群众安全感和满意度。抓好安全生产。牢固树立安全发展理念， 强化底线意识、红线意识和忧患意识，严格落实安全生产责任制，加强安全生产工作考核权重，突出抓好煤矿、道路交通、消防安全、食品药品等领域和行业的大排查、大整治、大防范，从严管控、从严处罚、从严追究，做到“全覆盖、零容忍、严执法、重实效”， 确保安全生产形势稳定向好。

　　办好“十件民生实事”。（1）完成18 个贫困乡镇“减贫摘帽”， 实施52 个贫困村“整村推进”。（2）“3 个15 万元”政策新增扶持微型企业3000 户、带动就业1.5 万人以上，建成2 个创业孵化示范基地，城镇新增就业10 万人，公益性岗位安排就业困难人员6500 人，完成返乡农民工就业创业1.5 万人。（3）新增10 所寄宿制学校，建设3.9 万平方米农村中小学学生宿舍，小学、初中生辍学率分别 控制在1.8%、2.8%以内，完成中职招生2.5 万人。新建、改扩建20 所乡（镇、街道）公办中心幼儿园，实现乡镇全覆盖。开工建设双戛高中教育城。（4）开工城镇保障性住房7840 套、建成8544 套。完成市中心城区城市棚户区改造专项规划，启动城市棚户区改造7330 套（其中市中心城区2000 套）。改造农村危房1.2 万户。实施扶贫生态移民1659 户6500 人。（5）城乡居民最低生活保障标准提高10%以上。城乡居民社会养老保险参保人数达到106.5 万人，60 周岁以上符合参保条件的28 万人实现应保尽保。（6）城镇居民基本医疗保险和新型农村合作医疗政府补助标准提高到320 元，城镇居民基本医疗保险基金最高支付限额提高到15 万元，新型农村合作医疗每位参合农民累计看病报销最高支付限额提高到30 万元。完成中心乡镇卫生院达标工程7 所、新开工5 所。开工建设凉都人民医院。新建乡镇计生服务站2 个、村计生服务室40 个。（7）解决26.82 万人农村人口饮水安全问题。新增通村油路（水泥路）1500 公里。新增公交车30 辆以上、出租车300 辆以上。新增815 个行政村通小型客车，农村通客车率达到80%。启动市中心城区城市轨道电车有关工作。实施市中心城区集中供热100 万平方米。建成市中心城区8 处以上人行通道，完成9 个农贸市场改造提升，建成12 个社区超市。（8）实施76 所农村幸福院。（9）新建21 个乡村学校少年宫、10 个农民文化家园。新建5 个乡镇农民体育健身工程和30 个全民健身路径工程。（10）启动1 个生活垃圾焚烧发电项目，基本建成地表水环境质量检测系统，实现空气质量自动监测站县城全覆盖，机动车 尾气监测“黄绿标”覆盖率超过90%，每月发布空气质量监测信息。开工建设市中心城区市污水处理厂（二期）工程。启动水城河综合改造工程。

　　各位代表！新形势、新任务对政府工作和自身建设提出了更新、更高要求。我们将按照省委和市委的安排部署，扎实开展好第二批党的群众路线教育实践活动，切实落实好“正衣冠、照镜子、洗洗澡、治治病”的总要求，深入开展“四找四转四进”活动，补精神之钙、除“四风”之害、祛行为之垢、立为民之制。我们将严守政治纪律、组织纪律，始终与中央、省委、市委保持高度一致，坚持发展为要、民生为本、企业为基、环境为重的工作理念，把政府各项工作办实、办好、办出成效，以务实高效的作风服务于民，以勤政廉政的形象取信于民。坚持依法行政，建设法治政府。自觉接受人大及其常委会的法律监督、工作监督和政协的民主监督，认真听取民主党派、工商联和无党派人士的意见和建议，充分发挥工、青、妇等人民团体的桥梁纽带作用。重视公众监督和舆论监督，加强政府行政效能监察和重大项目前置审计，强化行政执法和执法监督，规范自由裁量权。加强行政问责，抓不落实的事、问责不落实的人。坚持真抓实干，建设有为政府。按照“政府要理事、领导要管事、部门要办事，办成事、不出事”的要求，大力发扬“钉钉子”的精神，突出实干、快干、敢干、会干，把党委的决策部署细化为具体的项目和抓手，找准位置、负起责任，明确路线图、列出时间表， 做到胸中有想法、手中有办法，实实在在抓好工作落实，脚踏实地 抓出工作成效。坚持转变作风，建设效能政府。认真落实中央八项规定、省委十项规定和市委十项规定，切实解决“四风”突出问题，严格执行“约法三章”，着力解决好“庸懒慢浮贪”、“吃拿卡要报”、“冷硬横推拖”的问题，全力打好政府系统效能建设攻坚战。加快政府职能转变，进一步减政、减负、减支，以政府的工作效率换取企业的发展效益，以干部的辛苦指数换取群众的幸福指数。坚持廉政建设，建设廉洁政府。敬仰理想，敬畏纪律，敬重人民。认真执行党风廉政建设责任制，严格遵守廉政准则，强化廉洁自律，守住生活底线、工作底线，管好班子、带好队伍。深入开展“阳光晒权” 活动，把权力关进制度的笼子，严防工程建设、矿产开发、土地出让等领域的贪污腐败，切实解决以权谋私、与民争利和损害群众利益的问题，大力营造风清气正的政务环境。

　　各位代表！完成今年改革发展各项目标任务，实现对人民群众的庄严承诺，我们将义无反顾，勇往直前。让我们紧密团结在以习近平同志为总书记的党中央周围，在市委的坚强领导下，大力弘扬“奉献、包容、创新、超越”的新时期六盘水精神，进一步解放思想，开拓进取，凝心聚力，真抓实干，为与全省全国同步全面建成小康社会而努力奋斗！
